# Organisational structure configurations, their application and contribution to business performance in Greek shipping companies

**DOI:** 10.1007/s13437-023-00315-4

**Published:** 2023-05-30

**Authors:** Iraklis Lazakis, Robert Van Der Meer

**Affiliations:** 1 Department of Naval Architecture, Ocean and Marine Engineering, University of Strathclyde, Glasgow, UK; 2 Department of Management Science, University of Strathclyde, Glasgow, UK

**Keywords:** Organisational structure, Hybrid organisational framework, Greek shipping companies, Family-oriented organisational elements

## Abstract

The maritime industry is the cornerstone of the transportation of goods worldwide, offering safe, secure and environmentally friendly operations. Greek-owned and managed shipping companies and their successful performance can also be attributed to their unique organisational structure. The present paper explores the operational and theoretical organisational features in relation to Greek shipping companies and establishes to which extent these contribute to their business performance. Primary and secondary sources of information are considered while also employing semi-structured interviews and questionnaires. Two shipping companies each with a diverse fleet of vessels are examined through the development of 8 organisational structures and 12 company structural elements. It is found that Greek shipping companies’ structure is described by a hybrid organisational framework, combining the family-oriented strong leadership, internal company bonds, solid management, rapid adaptation to change and the attraction of loyal investors. Company A should further streamline the specialist organisational elements within operations (bunkering and chartering), technical and corporate functions (commercial, finance, training, IT, HR, insurance and legal) department while company B should upgrade the finance (accounts and invoice controls teams), HSQE (health, safety, environment, quality), operations (chartering element) and technical (purchasing, new building and performance teams) departments.

**Supplementary Information:**

The online version contains supplementary material available at 10.1007/s13437-023-00315-4.

## Introduction

The maritime industry is considered the cornerstone of transportation of goods worldwide, offering safe, secure and environmentally friendly operations The importance of shipping is denoted by the International Chamber of Shipping which suggests that more than 90% of the worldwide trade is conducted by ships while the international trade has been steadily increasing over that last 2 decades (ICS 2020). The importance of shipping worldwide can also be demonstrated by the main contributors per country in terms of owned vessels. In this case, Greece has maintained and increased its global leadership position (more than 17%) while Far East ship owing countries such as Japan, China, Singapore and Hong Kong follow with around 11.5%, 10.5%, 6% and 5% respectively UNCTAD (2020). In this case, it is interesting to note the continuous growth of the Greek-owned vessel capacity, while Chinese and Singaporean ship owners continue to increase their carrying capacity in an effort to establish transportation independency for carrying a variety of goods needed domestically.

At the same time, despite the merits that are present within the shipping industry globally, a number of challenges exist including the Covid-19 pandemic and its impact on maritime transport including a slump in exports and imports of goods worldwide and the effect of raising bunker fuels prices (Berti 2020). Moreover, despite the technological advances and application of strict maritime regulations, marine accidents constitute another major challenge the shipping industry is faced with (EMSA 2019). Further to the latter, the impact on the environment by ships’ CO2, NOx and SOx emissions together with accidental seawater pollution provide the grounds for additional hurdles that the shipping community is addressing on a daily basis (Raval et al 2019). Having the above in mind, a key question arising is associated with how the maritime sector and Greek shipping companies in particular manage to overcome such challenges and thrive globally. In the following sections, this is examined in the light of the companies’ organisational structure and the particular features of theoretical frameworks as demonstrated in the existing literature.

The present paper is structured as follows: Section 1 presents the introduction to the topic while section two critically examines the current literature related to the maritime industry’s key elements, operational methodologies and theoretical principles of organisational structures and application in shipping companies and in particular within Greek shipping companies. Section 3 describes the methodology and approach employed in this paper while Section 4 demonstrates the application of the described methodology in the case of two Greek shipping companies operating and managing a fleet of vessels in the wet/dry cargo sector (tankers and bulk carrier ships). Finally, Section 5 identifies and discusses the shipping companies’ cross-case analysis and interprets the key findings and finally provides an overall discussion on the above and concludes by suggesting a number of updates on the shipping companies structure together with recommendations for future work.

## Literature and critical review

In order to provide the context in which shipping companies are structured and operate, the fundamental regime of organisations’ structures and how these apply within the shipping sector will be discussed next. Hansen et al. (2020) explore the operational management plans available within shipping companies and how these can support crew operations onboard vessels. Related to the latter, Yuen et al. (2019) study the main factors for the development of the sustainable business performance of shipping companies’ management processes. In this case, internal company resources, relationship management and the company’s learning resources are the major influential factors for sustainable shipping operations. Although this is an expected outcome confirming similar theories applied in company organisational settings across other industrial sectors, it does not consider the dynamic operational environment of the shipping industry which may render the above theories challenging to apply within the sector. Son and Kim (2014) also discuss on the business process management method for allocating jobs for ship’s hull design and production while the development of an integrated process management system in ship management companies is investigated by various authors too (Celik 2009; Seo et al. 2018).

Chou and Liang (2001) assess the investment opportunities in relation to the performance of shipping companies by developing a model based on the application of Fuzzy AHP and MCDM in order to capture the inherent vagueness of information present in such conditions. Moreover, Panayides (2003) examines the competitive strategy-performance relationship within ship management companies suggesting that there is a positive relationship between pursuing competitive strategies and company performance in ship management. The study further suggests that achieving economies of scale, differentiation in a wider range of services offered and market-focus and competitor-analysis are the strongest influences on company performance.

Lyridis et al. (2005) discuss the business process modelling methodology applied in the case of a liner shipping company’s operations. The authors suggest a number of technology improvements for a specific voyage scenario which renders major time and cost savings leading to an increase in the number of round trips per year. Triantafylli and Ballas (2010) refer to the Management Control Systems (MCS) and how these may enhance the performance of shipping companies. The study suggests three categories of implementation including setting standards and supporting operations of the business; collecting information on cost minimization; and focusing on compliance with the requirement of cargo owners. The above application reinforces the high-quality operation of Greek shipping companies in conjunction with variables such as the experience of the person implementing the MCS, the size and age of the company. Tsouknidis (2019) also considers the relationship between personal/family and institutional ownership and its effect on firm performance for US-listed shipping companies. The findings suggest a negative relationship between the split on the percentage of institutional ownership and firm performance, which is primarily attributed to non-strategic decision fuelled by opportunistic investment behaviour rather than longer-term strategic options.

Having in mind the above, it can be concluded that although there has been an influx of research work investigating ship operational aspects, no such research has been referred to the combination of ship operations and a shipping company’s organisational structure and the effects such a combination may have. On the other hand, it would be beneficial to examine the theoretical domain of companies’ organisational structure with reference to the examination of shipping companies’ organisational structure highlighting their specific characteristics. The organisational structure can be defined as the internal setting and configuration providing a clear distinction among decision-making, distribution of tasks and targets set which will enable an organisation within a particular working environment to achieve its aims in addition to considering internal and external influences (Greenberg 2011). Mintzberg (1983, 1989) initially provided a clear definition on the types of organisational structures referring to the machine (hierarchical, bureaucratic, flat, formal and standardised make-up), professional (relying on highly competent experts), diversified (divisional, multiple functions/products), innovative (adhocratic, quick to change and adapt) and entrepreneurial (not structured, informal, lack of standardisation) structures, the missionary (standardisation of norms, ideological key element, decentralised) and the political (self-interest and informal power prevailing) organisational types. Galbraith (2009) expanded the above structure to include the matrix organisational structure in a combination of the machine and diversified ones.

In a similar manner, Bolman and Deal (2013) present their seminal work on internal organisational arrangement discussing on four types of frames: the structural (mechanistic level of arrangement), the symbolic (importance on company beliefs and values), the political (prevailing individual power and interests) and the human resource management one (management as a family, personnel key competences). Associated to the strictly structural frame, Taylor (2008) suggests the prevalence of strict hierarchy, technical competence, high level of formalisation and specialisation referring to the bureaucratic organisational structure. In a different setup, Willem and Buenes (2009) discuss the dimensions of corporate organisations comprising the coordination, centralisation, specialisation, formalisation and configuration elements. Lunenburg (2012) also refers to Mintzberg’s three main dimensions of organisational structure including the key decision-making part of an organisation, the mechanism supporting the coordination of tasks and the decentralisation type involving internal members to decision making.

Kuprenas (2003) also suggests the use of a matrix structure within organisations where a solid hierarchical structure is in place combined with the technical functionalities and support needed within a company. The latter strongly correlates with the structure mostly present within a shipping company having a strict functional organisational layout while at the same time different departments can combine knowledge and expertise to run a fleet of ships. San Cristóbal et al. (2018) explore the use of functional, pure and matrix types of organisational structures within a project management setting and highlight their advantages and disadvantages. Andersson et al. (2019) also discuss on the important traits that a company needs to possess in order to develop organisational resilience in the face of anticipation of external challenges suggesting that a company needs to view leadership and organisational structure as a continuous balancing act in order to seek internal stability. The latter is an important feature within the organisational structure of Greek shipping companies which through their predominant family-oriented and solid management and leadership setup, they apply agile, fast-enacting, and internal cooperation features.

Associated to the link between corporate governance and board practices considering Greek shipping companies, Koufopoulos et al. (2009) suggest that the majority of shipowning and management companies follow a family-based structure and protocol and thus are strongly influenced by a small leadership board advising on their decision-making process while the presence of external board members is negligible to non-existent, in agreement to previous studies by Harlaftis and Theotokas (2004). They conclude by suggesting that legislative pressure and the potential need for additional financial resources for the expansion of the company portfolio may lead the company to internal restructuring and openness to non-family members and executives, following more traditional corporate governance structures.

Theotokas (2007, 2018) also refers to the key elements and specific characteristics that are associated to the development of Greek shipping companies. He suggests a combination of the particular characteristics of the typical family-oriented hierarchical organisational structure and the development of a tacit knowledge base originating from years of practical experience both onboard ships and at the main office onshore. He elaborates further to also include the extensive trust and networking among key company stakeholders and the industry, the entrepreneurial philosophy in terms of being agile and adjusting to external threats and opportunities constantly present in the sector and the fragmentation of the Greek shipping business due to internal or external pressures. The latter complements what Stopford (2009) presents in a systematic overview of the structure of shipping companies which has evolved over the last decades. In this case, he refers to “the beneficial owner” company structure which includes one-ship companies, a holding company and a management company in an effort to derive organisational benefits and avoid liabilities in the form of high taxation, the latter also highlighted by Bergin (2015). This is complemented by the innovative practices of the Greek shipping industry which are elaborated and compared to the ones originating within the Norwegian shipping by Tenold and Theotokas (2013).

Considering the application of innovation within companies’ structure, Naveed et al. (2022) also elaborate on the effect of organisational resistance and how this applies within large organisations such as the banking sector. Their paper concludes that company improvement was shown in areas where the personnel was faster to adopt innovation aspects considering management of change as well. This comes in addition to the findings by Kim et al. (2023) who suggest the correlation between company positive innovation performance and organisational diversity within a company. Related to the above, the findings of both studies also reflect on the structure and positive performance of shipping companies which operate within a diverse environment including innovations suggested by national and international regulations as well.

Additionally, Canale et al. (2023) discuss on the application of ambidexterity and its effect on family firms. The paper suggests that family maturity and family social responsibility are two factors that drive the innovation exploration and exploitation of family firms and assist in the development of their dynamic capabilities while driving excellence; a particular characteristic and that is also predominant in family-run shipping companies no matter the size of their fleet of vessels they own/manage/operate. The effect of organisational ambidexterity influenced by the fast-changing environmental conditions is also supported in a study on the financial services sector by Yunita et al. (2023). The authors suggest that technological advances are even more present nowadays and affect the sector; an aspect which is also present within the shipping sector through the introduction of vessel performance measurement and compliance with international regulations on ship emissions and other environmental aspects too.

Related to the above, Lyridis and Papaleonidas (2019) also examine the structure of tanker shipping companies predominantly employing the hierarchical organisational structure built around key departments and personnel which are usually associated to the family-owned perspective. In this case, a clear structure of command, decision making and responsibility is evident to all employees and dissemination of communication and information is straightforward. On the other hand, this structure can become too rigid when applied in larger organisations and challenging to adjust in sudden changes within the sector. To overcome the latter, they extend their discussion by suggesting that a matrix structure may be employed in cases where more complexity is needed to operate and manage a wider fleet of vessels. They further discuss on the decentralised form of Business Process Management as mentioned by Bielic (2009) which improves the interaction among the company departments by introducing better communication and coordination of activities. This coincides with the merits and challenges of the matrix organisational structure as mentioned by Vantrappen and Wirtz (2016) which suggests the allocation of management duties to specific personnel while also encouraging the initiatives of other departments and personnel within the company. Decentralisation occurs in the form of a fleet manager who can combine the company’s centralised perspective applicable on the fleet element as well. On the other hand, typical challenges may include a loose internal control structure and while complexity may also be an issue when personnel need to go through multiple layers of a decision-making process. The above is also highlighted by a paper on the safety implications this type of structure may have especially when decentralising the organisational structure in big enterprises (Monteiro et al 2020).

## Theoretical framework

### Shipping company structure—internal and external actors

**Fig. 1:**
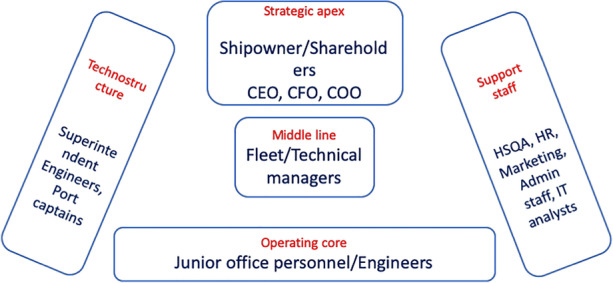
Typical Shipping company structure—internal actors coalition (source: authors’ own view adapted from Mintzberg 1989)

**Fig. 2:**
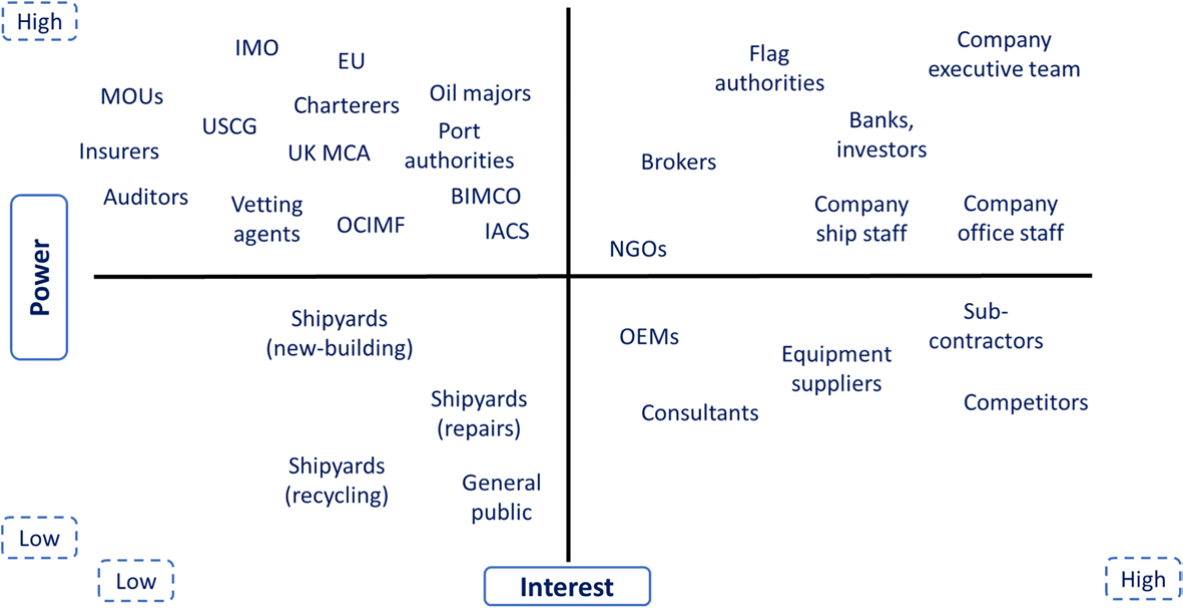
Typical shipping company power-interest matrix (source: authors’ own view)

Shipping companies assigned with the management of day-to-day ship operations face a challenging task as they need to include the optimal use and involvement of a number of stakeholders. The latter refers to internal actors such as the shipping company’s technical/financial/managerial personnel (Fig. 1) as well as external actors involving regulators, suppliers, insurers, industry bodies, consultancy firms and charterers among others (Fig. 2).

The internal actors’ coalition includes the Strategic apex actors such as the shipowner or shareholders of the company together with the company executive team (CEO, CFO, COO). Just below, the middle line involves the company fleet and technical managers, responsible for managing a number of ships within different fleets. These are supported by the technostructure including senior superintendent engineers and port captains who (usually) have experience and expertise from both the shore- and ship side of the company operations in order to deal with the company engineering and deck departments. On the other hand, the overall company structure is complemented by the support staff which comprises the HSQA, HR, marketing, administrative and IT analysts. Finally, the operating core involves the junior office personnel and engineers who deal with the day-to-day jobs within the shore side of the company structure.

Related to the external actors’ coalition shown in Fig. 2, a power-interest (P-I) matrix is a powerful tool which can depict and map the external stakeholders’ influence within a shipping company’s operations (Mendelow 1981; Olander and Landin 2005; Rosso et al. 2014). In this case, stakeholders are mapped in relation to their ability to influence the company strategy or project resources (power) and the extent they are interested in the company’s success (interest).

The above P-I matrix is presented in order to demonstrate the relative mapping of stakeholders in an effort to denote their grouping affecting the ships’ operations rather than mapping the specific power and influence characteristics of each one of them in relation to the shipping company structure. Having this in mind, the upper right corner of the matrix suggests that key stakeholders such as the company executive team, the onboard and onshore company personnel, flag authorities and brokers have a combination of relatively high power and high interest in the company benefiting and they should be clearly engaged. NGOs are also placed in this quadrant denoting the increased power they have over the last years. On the other hand, the top left quadrant provides a view of the stakeholders that have high power and are less keen on the shipping company’s interests. This group mostly includes the international e.g. IMO, EU, MOU authorities (PMOU 2020) and national regulators i.e. UK MCA, USCG, (UK MCA 2020, USGC 2020) and also industry players and associations such as OCIMF, BIMCO, IACS, charterers, oil majors, vetting agents, insurers and auditors (OCIMF 2017, IACS 2019). This high-power group needs to be also kept satisfied and informed on the company’s operations while not being over-consumed with the company day-to-day activities.

Supplementing the above, the lower section of the P-I matrix shows the least (to the shipping company) power stakeholders. This is subdivided into the high-interest ones such as OEMs, equipment suppliers, sub-contractors, consultants but also competitors in the shipping market which can be helpful when going through the detailed company operations. The low power-low interest players include the shipyards associated to ship building/repairs/recycling and the general public as well. These stakeholders could and should be informed of the company’s operations when required.

Considering all the above remarks, it can be deducted that, currently, there is no elaboration on a shipping company’s organisational structure and framework for managing the day-to-day ship operations. Combining the above with the volatile and highly unpredictable nature of the shipping sector, one needs also to consider that the vast majority of shipping companies manages such adverse conditions by a process which is based in a more of a word-of-mouth way, with senior and experienced personnel advising their younger colleagues rather than a thorough and well-structured approach based on existing theoretical frameworks which however have not been explored in the context of the shipping sector. In order to address the above, the contribution of this paper provides a novel contextual organisational framework and suggests the examination of a shipping company’s operations in the light of 8 different organisational structure configurations and 12 organisational elements is developed and presented in the following sections.

### Organizational configurations and shipping company structural elements

In this section, the novel organisational framework is described consisting of organisational structure configurations and shipping company structural elements presented in Tables 1 and 2. In this respect and to the best of the authors’ knowledge, no such framework has been presented in the existing literature before while the application of the framework in the case of the Greek shipping sector showcases another aspect of the paper’s novelty as well. In more detail, Table 1 shows the organisation’s structural configurations, merits, challenges and key theoretical association advised by the work of Mintzberg (1983, 1989), Taylor (2008), Kuprenas (2003), Galbraith (2009), Bolman and Deal (2013), as well as presenting the authors’ own view. The mentioned configurations are presented in terms of merits, challenges and key theoretical association.

**Table 1:** Organisation structural configurations, merits, challenges and key theoretical association (sources: authors’ own view considering Mintzberg (1983, 1989), Taylor (2008), Kuprenas (2003), Galbraith (2009), Bolman and Deal (2013))

Structural configuration	Merits	Challenges	Key theoretical association
Entrepreneurial	Informal, flexible, strong leadership, clear apex and line of decision making and communication	Strictly controlled strategy and operations, little space for flexibility and manoeuvring	Strategic apex
Machine	Clear hierarchical structure, specific line of responsibility across all levels of operations, simple, stable, precise, efficient	Control obsession, bureaucratic, internally- focussed, resistance to change, slow adapting to external pressure	Technostructure
Innovative	Organic structure, autonomy, dynamic, quick response to changes, high learning potential, divergent, effectively evolving	Inefficient at times, costly, ambiguous and fuzzy decision making, long time to operational effectiveness, less stable	Support staff
Professional	Decentralised, autonomous working cells/teams, highly trained personnel, collective decision making, stable	Complex, coordination issues, discretion misjudgement, difficulty to innovate, slow response to external pressures, time-consuming decision making	Operating core
Diversified	Clear allocation of capital resources, flexibility within divisions/teams, clear performance controls in place, independent operations within divisions/teams, internal training middle managers regime	Heavy on financial performance-based control, costly application (multiple teams), tendency to move towards Machine structure, overlook quality/human resource goals	Middle line
Missionary	Stable, strong leadership, clear focus, inspirational, distinctive, coordinated actions	Strong single-belief focus, issues of isolation, hard to observe external changes	Ideology
Political	Multiple independent perspectives, internal personnel development, potential lead to changes needed within	Strong self-interest orientation, discretion misjudgement, conflict issues, struggle for control of power	All aspects can be involved to some extent
Matrix	Specific line of responsibility, effective and clear performance controls, independent and flexible operations within divisions/teams, efficient flow of communication, openness, cooperation, flexible decision-making	Vague line of authority and accountability, internally-focussed, resistance to change, heavy on financial performance-based controls, costly application, overlook quality/human resource goals, competition for same resources	Technostructure, Middle line

**Table 2:** Organisational structure elements to be considered within the context of a Greek shipping company (sources: authors’ own view considering Theotokas (2007, 2018), San Cristóbal et al. (2018) Andersson et al. (2019))

Greek shipping company structural element	Features and narrative
Personnel involved	Type of personnel included in the company e.g. shipowner, Chief Executives, senior/junior personnel across different departments, non-executive stakeholders
Line of leadership and authority	Actors involved in line of command e.g. ship owner, shareholders, higher level decision-making personnel
Path of decision making and responsibility	Decision making sequence e.g. shipowner, Chief Executive suite, fleet managers, superintendent engineers/port captains
Personnel experience and expertise	onboard ship experience and expertise e.g. years of sailing experience, ship deck/engine department, number of university graduate/postgraduate/research degree holders (Phd, MPhil, MRes)
Division of teams/personnel	clear and specific division in fleet teams and departments within the company e.g., fleet 1, fleet 2 etc
Interdependency/interaction	degree of interaction and collaboration among fleet teams/personnel e.g. collaboration of personnel between fleet 1 and fleet 2
Internal competency development	Personnel participation in training courses/seminars, further education, e.g. development of onboard experience through ship new-building projects, junior staff sailing onboard ships to get relevant experience
Company culture	Loyalty and trustworthiness, confidentiality, good impression in the market associated to e.g. personnel retention indicators, company suppliers’ relationship
Risk mindfulness	Vulnerability assessment, proactive responsiveness, corrective actions and mitigation e.g. company risk prevention and mitigation planning
Flexibility/adaptation/agility	bottom up and top down across decision-making levels being proactive/responding to challenges e.g. responding to major turmoil (regulatory changes, pandemics)
External stakeholders affecting company	Influence of external actors on company overall decisions and operations e.g. IMO, international/national regulators, IACS, industry associations (OCIMF, BIMCO, Intertanko, etc.), insurers, vetting agencies, NGOs, competitors
External stakeholders being affected by company	Company influence on external actors e.g. charterers, suppliers, sub-contractors, shipyards related to company building/repair/recycling projects

The entrepreneurial structure presents the key merits of being an informal structure while flexible enough, including strong leadership, clear strategic apex and line of decision making and communication. On the contrary, it incorporates a strictly controlled strategy and operations leaving little space for flexibility and manoeuvring. The machine structure presents opposite characteristics to the entrepreneurial one and promotes the technostructure including a clear hierarchical structure, specific line of responsibility across all levels of operations while being simple, stable, precise and efficient. On the contrary, it can be too tightly controlled, bureaucratic, internally focussed, with resistance to change and slow in adapting to external market pressure. On the other end, the innovative structure consists of an organic and autonomous structure, being dynamic to respond to external changes, with high learning potential, divergent and evolving through challenges. However, at times it can be inefficient, costly, ambiguous and fuzzy in decision making, demonstrating long lead times to operational effectiveness.

The professional structure supports decentralised and autonomous working cells/teams, highly trained personnel and collective decision making. It is stable in its form; however, it can be complex, while may include coordination issues, decision misjudgement, difficulty in innovation which can lead to slow response to external pressures could originate in its full application. The operating core of junior engineers and personnel could favour this type of structure. Complementing the above, the diversified company structure encourages the middle line of personnel such as technical managers and superintendent engineers. This group is presented with a clear allocation of capital resources, flexibility within divisions/teams (a key aspect of the entrepreneurial structure), clear performance controls in place and independent operations within divisions/teams also supporting internal company training. This structure demonstrates heavy reliance on financial performance-based control and costly application when in multiple teams (similar to the innovative configuration) with a tendency to move towards the machine structure and overlooking quality/human resource goals.

Similar to the machine and professional structures, the missionary one provides a stable and strong leadership, inspirational and distinctive spirit, while also providing coordinated actions with a clear focus. On the contrary, it can be too self-focused, showing elements of isolation while being hard to observe and respond to external changes and provide applicable solutions in comparison to the innovative company structure. In this case, the political configuration which includes multiple independent perspectives, internal personnel development (similar to the professional structure) and the potential to lead in changes needed within the organisation, also incorporates a strong self-interest orientation, which can lead to discretion misjudgement, internal conflicts and struggle for control of power.

As a further division of organisational structure, the matrix configuration is presented as the one referring to the strategic apex and middle line company personnel and integrates elements from the entrepreneurial and diversified groups. The matrix structure includes a specific line of responsibility, effective and clear performance controls, shows independent and flexible operations within divisions/teams and allows for the efficient flow of communication among teams, openness, cooperation and flexible decision-making. The latter can though lead to a vague line of authority and accountability, show signs of resistance to change, rely heavily on financial performance-based controls, overlooking quality/human resource goals while competition for the same resources can be a point of conflict among teams in the company. On the other hand, contemplating on the work by Theotokas (2007, 2018), San Cristóbal et al. (2018) Andersson et al. (2019) as well as considering the authors’ own view, the 12 organisational structure elements including their main features and narrative to be considered within the context of a Greek shipping company are shown in Table 2.

## Research methodology

This section presents the paper methodology which is then applied in the case of two Greek shipping companies. A thorough questionnaire (shown in Appendix A) was distributed to company personnel followed by a number of semi-structured interviews conducted with company staff in order to formalise a methodological way of collecting valuable information as described and supported by a number of researchers (Andersson et al. 2019, Langley et al. 2013, Van de Ven and Poole 2005). This step was based on the level of expertise and experience of company personnel available to be interviewed having in mind the companies’ operational schedule. Interviewees originated from the operating core and middle line of the companies’ structure with an average range of experience of 14 years in various positions within shipping companies.

The above presentation of 8 organisational structures and 12 organisational elements will provide the canvas through which the Greek shipping companies will be examined in the following sections. Additionally, a number of company documents were considered including the company’s Safety Management System (SMS) manual and the company current structure. It has to be noted that each shipping company structure can be very detailed and may include an extensive number of different layers among all the mentioned organisational features which may render the company structure cumbersome to present and analyse further. In order to overcome such a challenge, all the key topographies and functions of all departments across each company are presented in a summarised form keeping in mind the key elements of organisational structure this paper elaborates on without compromising or missing out on any major company function. Following the above clarification, the next sections of this paper elaborate on the “before” and “after” state of the shipping companies examined having in mind the following sequence of steps:

1. Overview and presentation of existing shipping company structure with reference to the 5 key organisational features mentioned in the previous section of this paper (Apex, Middle Line, Technostructure, Support Staff and Operating Core).
2. Presentation and analysis of the information and insight achieved through the questionnaires and interviews while also discussing on the challenges each company is faced with
3. Comment on the company’s organisational structure having in mind the theoretical organisational structure frameworks presented
4. Propose and present changes and updates within each company structure

It is also important to highlight the various specific features of the shipping company structure. Apex refers to the highest level of decision making and command including the ship owner and executive suite; middle line provides the link between the senior and lower level of structure within the company; operating core suggests the team members within each company department supporting each department; technostructure includes the specialist departments within the company (e.g. technical department, operations department) while the support staff involve personnel which provide their services throughout the entire company structure (e.g. finance, IT, HR). Moreover, the validation of the methodology and case studies results were conducted through discussing with the relevant shipping company personnel the original data derived from, highlighting the applicability of the suggested organisational structure framework. The above validation of the suggested organisational re-arrangement was conducted through semi-structured interviews with the organisations’ personnel from the operating core and middle line of the companies’ structure. Initially, the methodology structure and process were presented to them in detail while the results were then suggested too. It has to be noted that access and acquisition of data and associated information is a well-known challenge within the maritime sector, also considering the confidential nature of such information. Nevertheless, following the authors’ efforts, data were retrieved and analysed and are presented through the application case studies shown in the following section.

## Case studies and results

In order to apply the above methodology, two different companies were selected which operating/managing tanker and bulk carrier ships (Table 3). Additional information on tanker and bulk carrier vessel definitions are shown in Appendix B.

**Table 3:** Company A and B’s key features

Characteristics	Company A	Company B
Fleet size	30	32
Type of ships	Crude oil tankers (VLCC, Suezmax, Aframax, MR1)	Bulk carriers (Capesize, Kamsarmax, Panamax)
Total dwt carrying capacity (million tons)	5.0	4.4
Total number of seafarers employed	750	800
New building projects	Aframax tankers (3)	Bulk carriers (2)

**Fig. 3:**
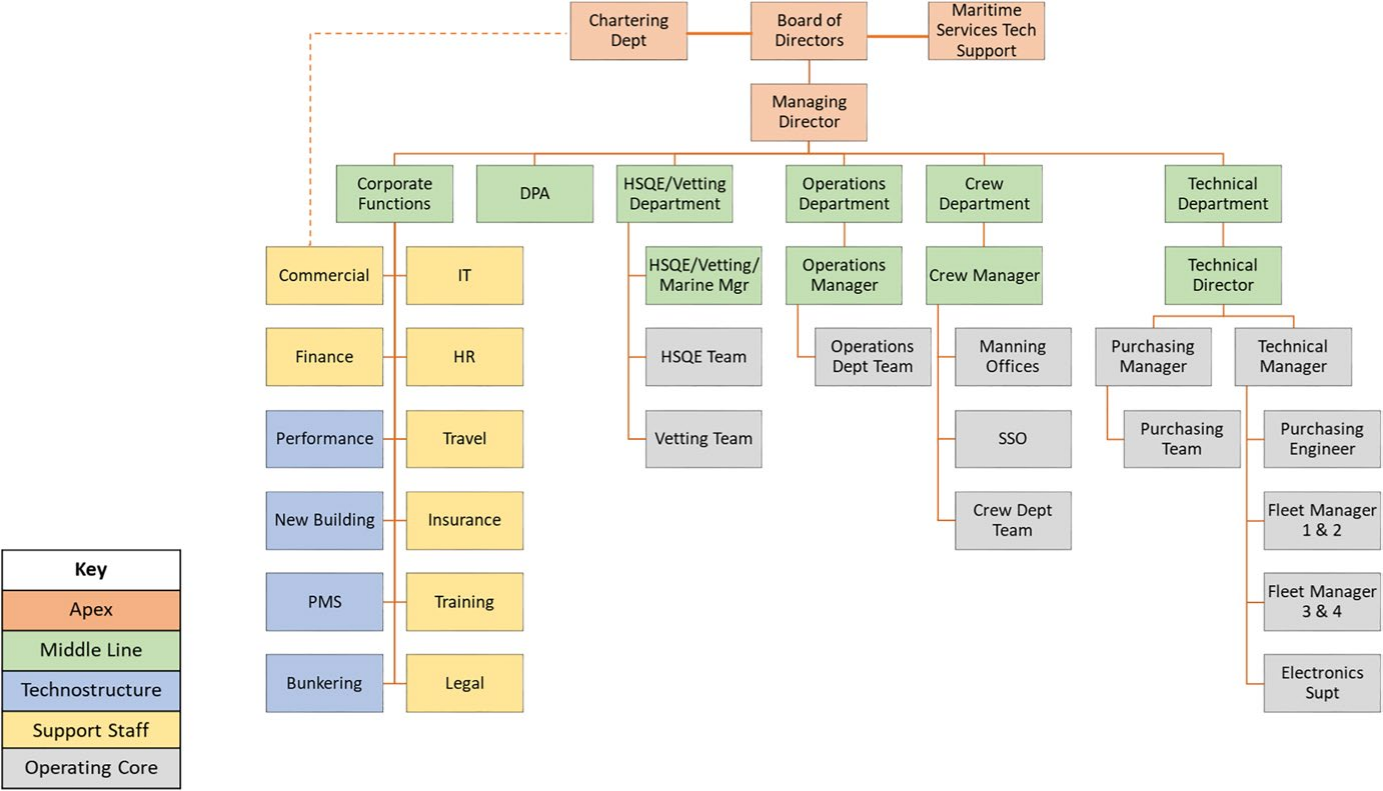
Company A current organisational structure (source: author’s own view)

Company A manages a fleet of crude oil and oil product tanker ships, while company B operates a fleet of medium to large bulk carriers. In line with the company’s internal actors’ coalition presented in Section 3, the various company features and departments are highlighted using different colouring patterns i.e. Apex (orange), middle line (green), Technostructure (light blue), Support staff (yellow) and Operating core (grey) shown in Fig. 3. More information on the definitions of shipping company roles and departments are shown in Appendix C.

The Apex element of company A includes the board of directors, the chartering department and the maritime services technical support. This is followed by the middle line of the hierarchy including the corporate, DPA, HSQE/vetting, operations, crew and technical departments. Company A organisational structure is complemented by the operating core functions such as the various teams present within each middle line department including the HSQE and vetting team, the operations department team, the manning offices, the shipboard safety organisation (SSO) and the crew team.

It is worth highlighting the technical department operating core element due to its importance in the day-to-day running and up-keeping of the company vessels. This includes the purchasing and technical manager, each of which manages the purchasing and technical teams comprising the fleet managers for a fleet of vessels (fleets 1 and 2 and fleets 3 and 4) and the electronics superintendent engineer. Additionally, the support staff element incorporates corporate functions such as the commercial, finance, IT, HR, travel, insurance, training and legal departments. Finally, the technostructure involves the ship’s performance, the ships new building, the PMS and bunkering elements as part of the existing corporate middle line of the company structure.

At the same time, being a tanker operating and managing company, it is engaged in a very strict regime of national and international regulations and guidelines that need to be followed in relation to safety, security and environmental protection. In this case, a particular example is the major expansion of the company HSQE department over the last 5–7 years from a single initial team of 2–3 personnel to the introduction of various teams and departments such as the vetting, marine, DPA and TMSA ones. In this case, it has expanded to include separate HSQE and vetting teams consisting of ex-captains and ex-chief engineers.

The path of decision making and responsibility is supported within each individual department (separate teams for the HSQE, crew, operations and technical ones), and there also exists some form of independent decision making for up to usually a certain financial amount. The importance of decision making and its conjunction with the level of hierarchy is also related and affected by the oil cargo market condition. In this case, better market conditions allow for more flexibility in decisions made, especially related to capital investment associated to technological advances i.e. onboard installation of scrubber system for control and reduction of ship emissions, sensors installed on key ship machinery and data acquisition and processing systems. This is supplemented by the company’s best practice of retaining key officers (captains and chief engineers) who have demonstrated an excellent profile on board the ship and who can then be drawn to the main office for further employment. In relation to the above, the level of the personnel experience achieved on board ships is supplemented by further education depicted by postgraduate (MSc level) studies along with additional training provided both internally and through external consultants.

Following that, although the formal distribution of responsibilities and authority is achieved through the various individual departments, the front line of command is mostly driven by the finance, HSQE, technical and operations departments which deal with the day-to-day running of the vessels (bunkering and fuel consumption, spare parts). Moreover, the key drivers for change attending to the market conditions are performed by the technical team in conjunction with other departments. A good example is provided by the queries on ordering and installing the scrubber systems for ship emission reduction, a major investment in the range of 1.5 million US$ per ship. The technical and purchasing team seek offers from three different makers providing the technical ship specifications as given by the technical and HSQE teams. Finance also advises on acquisition options and financial implications in the short and longer term.

The above example shows the cross-cutting collaboration both among different departments e.g. technical and operations, operations and finance and within each department separately e.g. technical and purchasing managers working under the technical director. The formal and informal company culture provides for the smooth operations on a daily basis while a future investment portfolio of new building ships in Far East shipyards in order to increase the company fleet capacity is currently under way. With regard to risk mindfulness, the company is aware of the new regulations and current market trends. Following the previous example of the scrubber system installation, it has proceeded in minimising the financial risk and technical risk by timely consulting with its competitors in the Greek shipping community, watching the market trends and be able to address these risks by opting for a limited installation of scrubbers on a number of ships combined with using high sulphur fuel oil on the remaining of the fleet. This has proved a successful and profitable decision as the fuel oil price in the latter case has drastically reduced during the same period compared to the use of low-sulphur fuel oil (Ship & bunker 2020).

In terms of its agility and adaptation to market changes, the company benefits by the Greek shipping community experience, expertise, extensive networking and strategic look into new technologies such as alternative fuels for the ships’ main propulsion (e.g. LNG, ammonia) while also using and maintaining online databases accessible to the technical department. On the other hand, external stakeholders affecting the company’s operations and organisational structure include the national and international regulators (IMO, EU, MOUs). Market conditions affecting the ships’ charter rates provide a fertile background for competition and/or cooperation with other shipping companies, together with other external factors such as NGOs, ILO, OCIMF, EMSA and port state control authorities (USCG, UK MCA) as seen in the power-interest matrix developed in Section 3. In conjunction with the above, the external stakeholders that are being affected by the company include the shipyards contracted for new buildings/repairs, while also equipment manufacturers and suppliers are prominently affected when ordering of spare parts together with the classification societies monitoring the ships’ technical aspects. Moreover, the ships’ fuel oil bunkering agents are also influenced by the company through its bunkering department.

The above-multifaceted characteristics suggest that although company A operates smoothly and manages to overcome and adapt to market changes, it also consists of areas that currently present to be challenging. In this case, it seems that the corporate functions middle line of management includes too many discrete and fragmented departments e.g. commercial together with finance, IT, HR and travel, other more technically oriented departments such as the performance, PMS, new building going hand-in-hand with operational ones (i.e. bunkering).

**Fig. 4:**
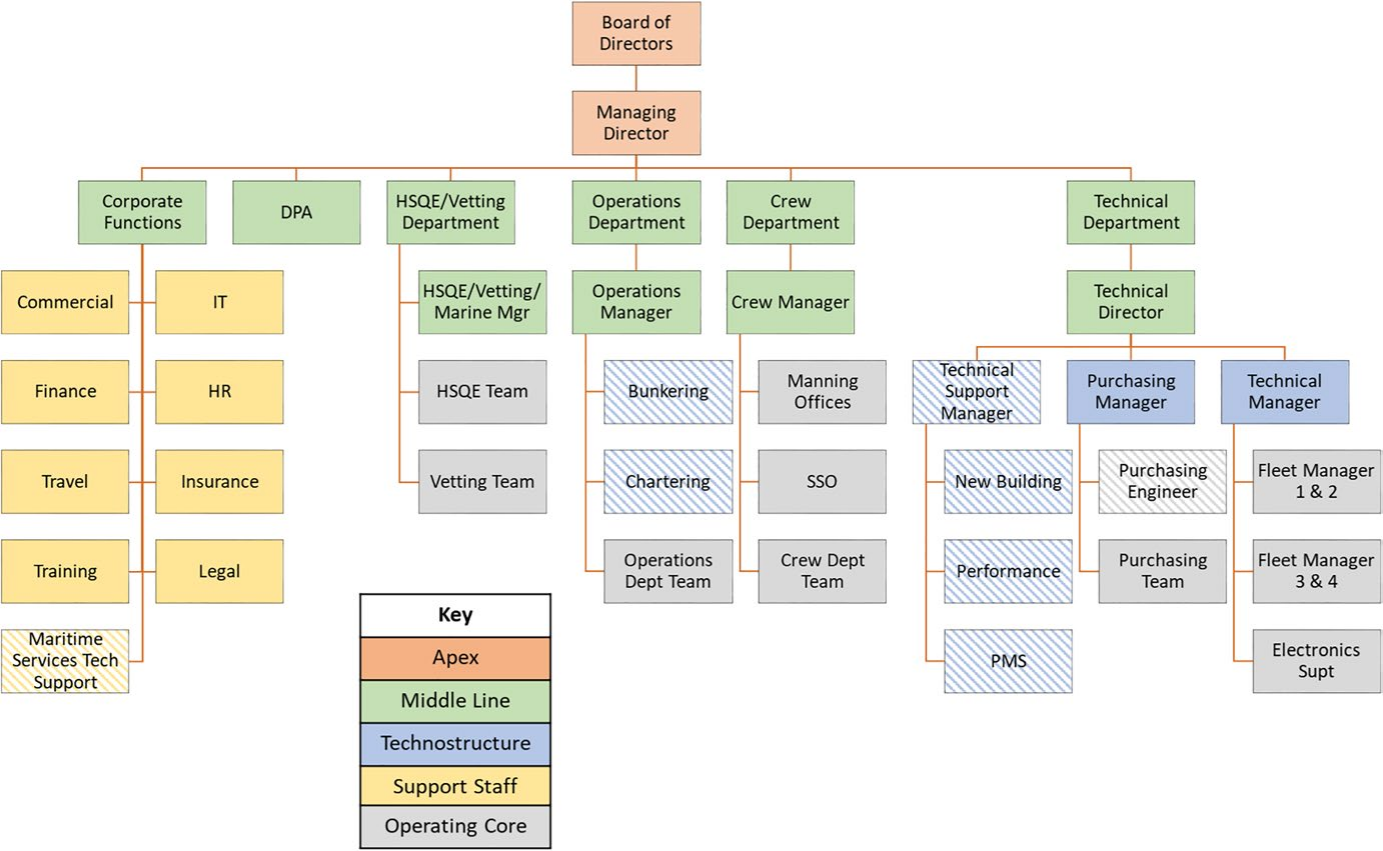
Company A proposed organisational structure—diagonal pattern suggests introducing new/moving specific role within a company department (source: author’s own view)

On the other hand, the chartering department seems to be directly linked with the highest of authorities such as the board of directors while the purchasing manager and team report directly to the technical director. The above combination of merits and challenges suggests that company A follows a hybrid organisational structure for which a number of updates in order to streamline its organisational structure can be found in Fig. 4.

The proposed updates include the transfer of the maritime services technical support from the Apex to the corporate functions in order to streamline the entire line of maritime services technical support throughout the fleet of the company (a typical example of support organisational structure). Moreover, the bunkering (provision of fuel and lube oil) and chartering (arranging and managing the chartering of the company vessels) teams have been moved within the operations department as they form an essential part of the day-to-day ship operations. Similar to the latter, the technical support manager is a new role which has been developed to incorporate the new building, performance and PMS teams as part of the overall technostructure of company specialists responsible of the technical management of the company fleet. In addition, the purchasing engineer has been moved from the technical to the purchasing manager to have a direct line of communication between managers within the technical department as well. In this respect, the purchasing manager and engineer will now be able to directly communicate and collaborate with the technical manager and feet of ships when new spare parts need to be ordered for specific ships, thus minimizing the time lost to verify and approve new orders as well as enable the quality control within the technical department overall.

**Fig. 5:**
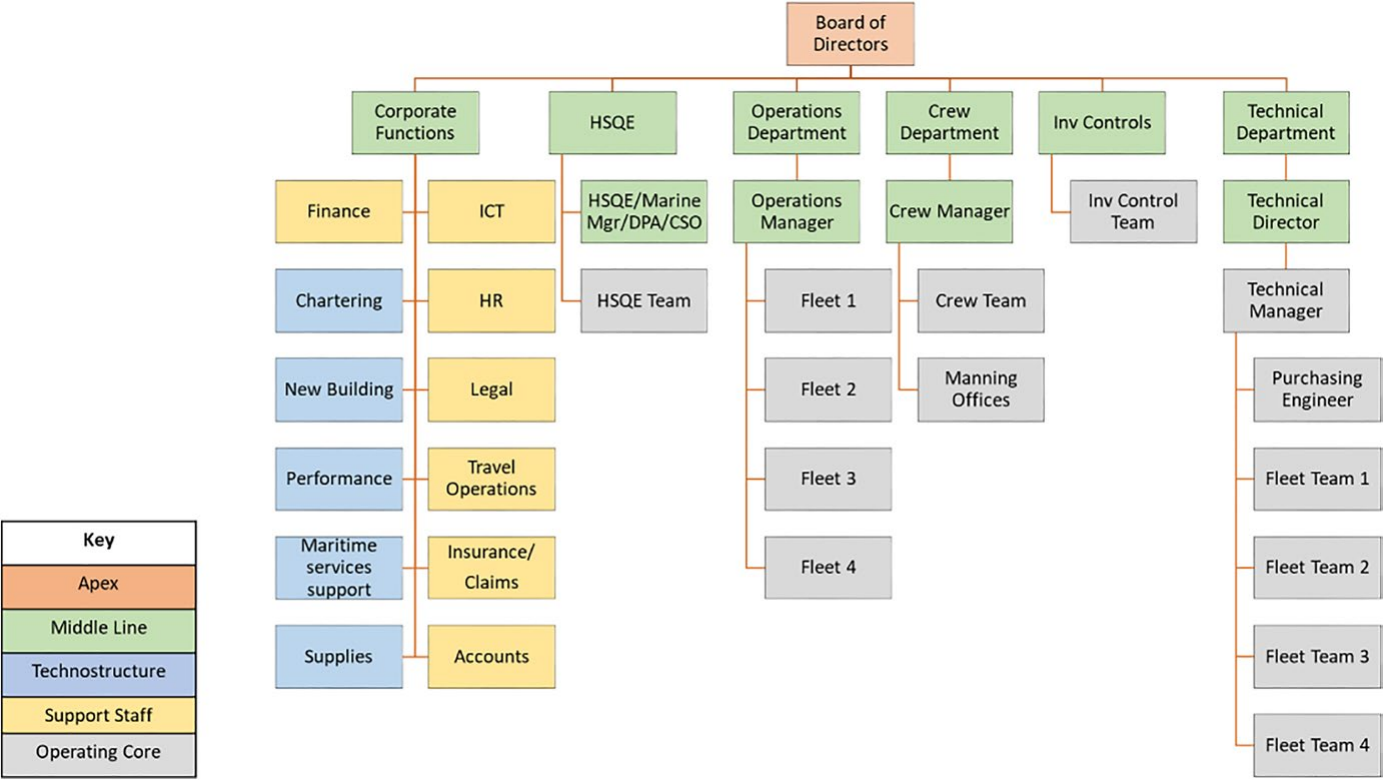
Company B current organisational structure (source: author’s own view)

Company B employs an organisational structure as can be seen in Fig. 5. The Apex line of command is provided by the board of directors including the CEO, CFO and COO. A second level of the hierarchy is delivered by the corporate functions department, the HSQE (with the HSQE, Marine, DPA and CSO managers), operations, crew, invoice controls and technical departments and respective managers which form the middle line of company management. These are complemented by the operating core structure which is present in the form of the HSQE team, the four different operations teams, the crew team, the invoice control team and the technical manager and fleet teams as well. It is worth highlighting that a purchasing engineer is aligned to support the technical director as well.

Additionally, the corporate functions department includes a number of support staff such as the finance, ICT, HR, legal, travel operations, insurance and claims and the accounts departments. Moreover, the specialist technostructure line of organisational structure incorporates a number of departments such as the chartering, new building, performance, maritime services support and the supplies departments. As can be seen, the overall structure is concentrated within a very diverse corporate functions group which includes several specialist (technostructure element) and additional support staff departments as well. The overall line of command and a higher level of management are provided by the ship owner and the executive team.

These account for major decisions such as the chartering of vessels and the ordering and building programme of new ships after internal consultation with the respective departments (i.e. technical, operations, finance, chartering). The latter also demonstrates the degree of communication and interaction between individual departments within the company in the day-to-day company operations.

Further on, company B crew and HR department have established a scheme in order to retain the best performing key ship officers who can be further trained in order to be drawn to office staff as senior managers such as port captains and superintendent engineers. The experience and expertise of such personnel are supplemented by office staff with high level of undergraduate and postgraduate degrees in addition to training provide by the company through both internal sources and by engaging external consultants. Within the line of responsibility, the finance and HSQE departments are followed by the technical, operations and chartering departments reflecting the level of decisions made especially when market conditions are at high levels. In this respect, the building programme of new ships is coordinated by the corporate functions although technical expertise and judgement are required as well.

Especially in the case of new regulations and industry trends, the company shows agility and adaptation by closely following the relevant industry standards and best practices, particularly considering capital-intensive investments e.g. scrubber systems installation onboard ships, live and online information collection systems including further data processing. It is important though to mention that although the bulk carriers shipping sector complies with the IMO and national/international regulations, these are not as stringent as within the tanker sector per se. On the other hand, the networking activities and consultation with competitors within the Greek and international shipping market allows for taking steps towards financial and technical risk mitigation and prevention. A good example is the ordering and installation of water ballast treatment systems onboard ships in order to comply with the latest IMO regulations. A consultation process was initiated with suppliers and shipyards while also having in mind existing industry practice and lessons learnt from similar systems being installed on competitors’ ships.

**Fig. 6:**
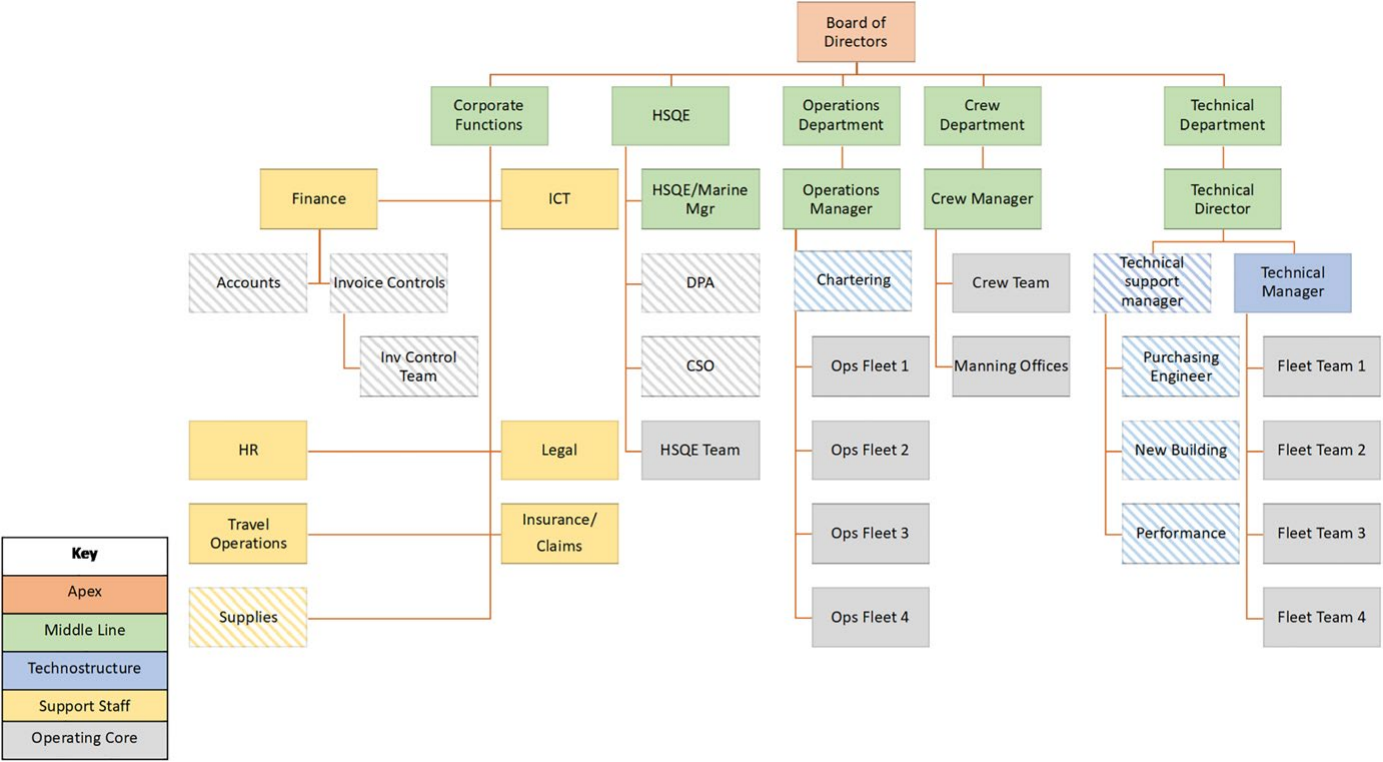
Company B proposed organisational structure—diagonal pattern suggests introducing new/moving specific role within a company department (source: author’s own view)

Moreover, due to the inherent differences and level of less complexity of the dry cargo sector compared to the oil cargo one, there is an informal company culture to proceed with more cautious steps when it comes to the retention of shipboard personnel (ex-captains and chief engineers) versus freshly educated and trained personnel. The latter also refers to the influence of external stakeholders on the company, such as the national and international regulators i.e. IMO, EU, port state control authorities (USCG, UK MCA) together with other external factors such as ILO and dry cargo charterers as seen in the power-interest matrix presented in the previous chapter. Additionally, the shipyards at which the building of new ships will be built together with the spare parts supplies, equipment manufacturers, classification societies monitoring the ships’ technical aspects and bunkering agents are directly affected by the company’s decisions. Having described the above structure and challenges involved in the daily company operations, Fig. 6 suggests the needed updates to streamline the organisational structure of company B.

As can be seen, the top level of the organisational structure includes the main board of directors (Apex element). Within the middle line of command, the structure has remained very similar; however, the invoice controls department has been moved from this level into the finance operating core together with the invoice controls team to support the overall company finances. In this case, the invoice control department will deal with the day-to-day accounts and invoices of the spare parts and equipment that are needed onboard ships and which require the control and approval by the technical department. This will improve the line of communication and collaboration between the two departments and will further facilitate the verification and validation of the ordering of spare parts and avoid the wrong parts sent onboard the ships with further logistical and financial implications.

Further discussing on the support staff element, the supplies department has been also moved to the corporate functions group as it oversees the overall company supplies, especially the ones sent to the dry cargo ships. The HSQE (middle line) department is also now supported by two additional operating core departments (DPA and CSO) which will be separately coordinating the communication with the company ships as a first-point-of-contact in case of an emergency (e.g. accident, pollution incident).

Furthermore, the technostructure elements within company B will be supported by the chartering department which is to be placed under the operations department coordinating the chartering of vessels to major dry cargo charterers. Additionally, a separate department has been created to fit within the technical department and sit next to the technical manager. This new specialist technostructure department accommodates the need to better coordinate the activities of all the additional technical activities of the company such as the purchasing engineer functions, coordinating the company’s new ship-building programme and also overseeing the performance of the entire fleet of vessels, functions which are too specialised to be included within the overall corporate department.

Performing the above updates, company B surpasses the challenges of communication among departments which may have been hindering the optimal day-to-day operations and allow for more flexibility and agile collaboration within the dry cargo shipping sector. It also provides a direct and prompt collaboration between departments e.g. when spare parts need to be ordered. In this case, when a requisition is made by a ship, the purchasing engineer is informed, then the technical superintendent initially approves the requisition followed by the technical manager. They on the other hand, return with a confirmation to the purchasing manager who notifies the purchasing operator and furthermore the purchasing engineer who arranges to send the correct spare parts to the particular ship. All these functions now sit within the technical department and the technical director thus avoiding miscommunications, at which case the spare part would not be delivered or the wrong one would make its way to the ship.

## Discussion and conclusions

In this paper, the main organisational theoretical frameworks were investigated together with the most recent updates including the shipping companies’ organisational theories and applications. From the above, it is evident that there is no direct association to one specific theoretical organisational structure within Greek shipping companies, moving clearly away from the typical family-oriented structure observed by Tsatsoulis (2010). This leads to the next major finding: there is no in-depth investigation on the Greek shipping companies’ structural features, having in mind the taxonomy suggested in this paper.

Having the above main findings in mind, the two shipping companies’ case studies present some common features in their organisational structure worth of further discussion. Overall, they both follow an entrepreneurial type of structure led by the strategic Apex consisting of the ship owner and the executive suite of directors. The merits of the entrepreneurial setup are clearly visible including strong leadership as for example in the case of ordering and financing new ship building projects in shipyards around the world; a clear line of decision making and communication; while also showing some level of flexibility especially when considering technological and regulatory changes such as the introduction of scrubber systems onboard ships for the control of emissions generated. The above resonates with what Theotokas (2007) and Pallis (2007) also refer to as the cornerstone of the effective management of Greek shipping companies which includes the combination of entrepreneurship, effective global market networking and an instinct for industry updates and changes which has assisted in the companies’ flourishing for more than a century.

On the other hand, however, it has been shown that this type of company structure may be rigid at times, with tight controls in place and little space for flexibility particularly observed in the sequence of budgeting allocation for spare parts and equipment to be sent onboard ships (direct decisions made by individual departments are limited to a few thousand US$) with slightly higher budgeting control and approval needed in most cases. This also brings in mind the machine type of structural configuration which although it may provide a clear hierarchy and specific line of responsibility needed in major decision making (e.g. major investment on scrubber systems installed on all the company ships), it tends to become bureaucratic in functionality, too internally focused and slow at adapting in external changes (Taylor 2008). However, the above machine structure characteristics have been essential when careful consideration on the use of technologically advanced tools for ship optimal routing, machinery sensors installation and data collection on ship performance monitoring and installation of scrubber systems and water ballast treatment systems was performed also considering the significant capital investment required.

At the same time, the mentioned shipping companies presented elements of clear allocation of capital resources among all company departments (corporate functions, HSQE, crew, operations and technical ones); clear performance controls in place (each department has its own specific team and performance metrics) and independent operations within each team (each department has its own manager and clear division of tasks). All the above provide a clear reference to the diversified organisational configuration as described by Mintzberg (1983) and Bolman and Deal (2013). This favours the middle line of the company hierarchy consisting of the various department managers which paves the way for internal training at higher positions and increased potential for promotion opportunities. It also demonstrates the key features of the diversified structure including a degree of flexibility within each department (operations, technical) and political one with its strong self-interest orientation and power control struggle (Kuprenas 2003).

The last observation also aligns with the matrix structural configuration showing the clear hierarchy, a specific line of responsibility, effective performance controls and shows that companies are heavily oriented towards the technostructure of theoretical association (Mintzberg 1989; Galbraith 2009). In this case, departments such as the HSQE, crew, operations and technical ones provide the specialist knowledge and expertise needed for the companies’ functioning. This is evident in the case of the collaboration of the technical, purchasing, operations and finance (corporate functions) department for the ordering and provision of the correct and of the right quality spare parts and equipment for the two companies. Another example of the above was shown in the internal communication and cooperation of the HSQE and operations departments for the chartering and vetting control and audits of the company ships by Shell/BP oil majors (company A) and dry cargo charterers (company B).

Moreover, both shipping companies also incorporate the elements of the professional configuration which consists of individual teams within each department. This is the case of the companies operating core of personnel which employ the HSQE (health and safety and vetting teams), crew (crew and manning offices team), operations (operations fleet teams) and technical (ship fleet teams). These rely on the operating core of a mix of personnel such as fleet managers, superintendent/purchasing engineers, training personnel, marine operations engineers and port captains. A key finding across both companies was that the operating core of employees is willing to accept that not everyone is equally reimbursed in terms of salary and rewards; however, there is a need to feel acknowledged for the effort they provide and they highly evaluate the additional benefits they may be offered such as company days off, internally arranged lunch breaks among others thus maintain a sense of fairness through financial awards or other benefits. The latter is also suggested by Querbach et al. (2020), Oraman et al. (2011) and Murphy and Simon (2002) who discuss on the added value of intangible benefits that a company may derive from its employees’ satisfaction. This is also signified by Thanopoulou (2007) who supports that the personnel contribution is a key factor in the rise and sustained progress of the Greek shipping companies.

On the other hand, a key difference in relation to the discussed corporate structures was present when investigating the details of the innovative configuration which is based on the support staff elements of autonomy, quick response to changes, high learning potential, divergence and evolving effectively (Fonseca et al. 2019; de Mello et al. 2012). In the case of Greek shipping companies support staff include the essential (although quite different to the current literature on organisational structures) areas of corporate functions departments such as the finance, HR, legal, insurance, claims, IT and training entities. These showcase elements of the operating core theoretical association which relate to the professional structural configuration; that is, demonstrating autonomous working teams/departments, highly trained personnel, stable structure and collective decision making within each department (Mintzberg 1983).

Summarising the key discussion and conclusions the strength and success of the Greek shipping companies mentioned above are provided through a mix of organisational configurations which render them in a unique formation of a hybrid pattern not seen in other industries before. The latter allows Greek shipping companies to thrive for a number of reasons. In this case, they can optimally combine the typical characteristics of family-oriented companies with the ship owner and/or key family members/relatives/colleagues positioned at the executive suite providing very strong leadership and internal company bonds, solid management with similar interests and no need for costly formalised processes for agents/managers within (Mitchell & Meacheam 2011), although the latter has been challenged lately (Li and Zuo 2020).

The hybrid structure also allows Greek shipping companies to adapt to rapid changes in the highly competitive maritime industry and attract loyal investors who can support further innovation (Zybura et al. 2020). As has been seen through the case studies, there is a high degree of transparency in the flow of communication while collegiality and trust is engraved among company employees (despite some issues present as shown through the interviews), which is another characteristic of family-oriented companies and commitment which leads to strong company performance (Allen et al. 2018). A similar level of trust is also demonstrated by external stakeholders such as suppliers, regulatory and industry bodies among others who are encouraged by the fact that they deal with the same company governance in the long run. The good company reputation developed over the years is specifically demonstrated in the case of investors and banking institutions which provide the capital funding for the company ship new building projects and technical innovations.

The longer-term perspective of the hybrid structure of family-oriented Greek shipping companies also allows them to be less exposed to financial risks, opting for lower share profits to shareholders but more robust and long-term results, which can then initiate capital investment and the expansion of their fleet and entry to new markets (e.g. LNG/LPG vessels). Furthermore, the hybrid company structure can be easier to benefit by political and governmental influence, supporting their overall decision-making and portfolio as discussed by Duran et al. (2017). Having this particular structure in place, the company can have direct control over finances thus differentiating from other sectors with more of a managerial corporate organisational structure (Carney 2005).

The above hybrid configuration also allows for a fine balancing act between the above-mentioned merits and particular challenges of family-oriented companies such as the confusing boundaries in between private ownership and agency issues (Schulze et al. 2001); developing symptoms of favouritism which may affect recruiting and promotion of the right person at the right position (Firfiray et al. 2018); and eventually managing a fast developing company which may grow out of the direct control of the initial founder especially when it comes to sharing the power generated with “new-comer” managers which are hired to run parts of the company as this continues to expand (Cadbury 2000). Having described the above, the specific companies suggested updates and recommendations are discussed next.

Moving forward, a number of changes and recommendations for the two shipping companies are proposed. Starting with company A, the major elements of internal restructuring suggest the streamlining of the corporate functions middle line of the company structure by relocating the technostructure specialist elements (performance, new building, PMS and bunkering departments). These are now placed under the operations department (bunkering and chartering) led by the operations manager and the technical department (performance, new building and PMS teams) supervised by a technical support manager. Moreover, it is further suggested for the technical department to incorporate a purchasing manager and team to enable the coordination and interaction among the ordering, purchasing and quality control of spare parts and equipment to be sent onboard the company ships. Additionally, the corporate functions department now includes the more generic but essential elements that apply and assist throughout the company i.e. the commercial, finance, travel, training, IT, HR, insurance and legal departments.

Related to company B, a number of recommendations apply across the company structure. In this respect, the finance department within the corporate functions Middle line of the hierarchy can include the accounts, invoice controls and respective team. Such a move will allow for all the company and fleet-wide financial transactions to be run within one department instead of dispersing them across other departments. Moreover, the supplies element can now be introduced as part of the corporate functions department, supporting the entire company’s needs. Regarding the HSQE department, two new roles are suggested: the DPA and CSO ones which can assist with the daily transactions and communication with the ship’s crew as a first point of contact in case of an emergency. The chartering element is suggested to be included within the operations department for optimal and direct collaboration with the company’s operations manager and the company fleet. Moreover, it is suggested for the technical department to be supplemented with the technical support manager sitting under the company technical director. Their role will be to coordinate and oversee the efforts of the purchasing engineer, new building and performance elements as these are directly related to the core aspects of the technical department and still provide the specialist knowledge in the area of ship new building projects and monitoring of the existing vessels daily routine.

Having performed the above research on Greek shipping companies’ organisational structure and operations, a number of suggestions for further study can by proposed. The present research may be expanded by including a number of additional shipping companies which own, manage and operate different types of vessels e.g. the fast-developing sector of LNG carriers, container vessels of all sizes, passenger ferries and roll on-roll off ships employed within the Greek domestic market. Moreover, it would be beneficial for future research studies to consider a wider spectrum of shipping companies i.e. the case of smaller size ones of up to 5 ships and larger ones with more than 50–60 vessels being owned/operated. Furthermore, it would be helpful if more company personnel were included while additional questionnaires were being returned across all levels of seniority and departments within each company in order to capture additional personnel expertise. The latter could be supplemented by also investigating the external actors which affect or being affected by the organisational structure of a shipping company as seen in the P-I matrix presented in the previous chapters such as charterers, insurers, ship personnel and supplies among others.

## Supplementary Information

Below is the link to the electronic supplementary material.

Supplementary file1 (DOCX 30 KB)
