# Supplementary material for: Organisational structure configurations, their application and contribution to business performance in Greek shipping companies
Source: WMU J Marit Affairs. 2023 May 30:1–28. Online ahead of print. doi: 10.1007/s13437-023-00315-4 (PMC10228423; doi:10.1007/s13437-023-00315-4)
Supplement: Supplementary file 1 — Supplementary file1 (DOCX 30 KB) [file 13437_2023_315_MOESM1_ESM.docx]

# Appendix A – Questionnaire distributed to Greek shipping company personnel

1. What type of personnel is involved within the company?
2. What is the line of leadership and authority?
3. Which is the path of decision making and responsibility?
4. In your view what is the current company organisational structure e.g. entrepreneurial, machine, matrix, etc.?
5. What is the personnel experience and expertise?
6. What is the division in teams/personnel?
7. Are there areas of interdependency and interaction?
8. What is the internal competency development?
9. What is the existing formal and informal company culture?
10. Is the concept of risk mindfulness present within the company?
11. What is the level of flexibility, adaptation and agility in comparison to industry, market and regulatory changes?
12. Which departments are more important within the company?
13. Which company departments/stakeholders drive the change within the company?
14. How does the company cope with change and new regulations/technologies/market updates?
15. Which external stakeholders affect the company the most?
16. Which external stakeholders are being affected by the company the most?

# Appendix B– Definitions of tanker and bulk carrier ship types

| **Acronym** | **Full definition** | |  |
| --- | --- | --- | --- |
| Aframax | Crude oil tanker ship size equal to 80-120k tonnes deadweight carrying capacity | |  |
| Capesize | Bulk carrier ship equal to 160-180k tonnes deadweight cargo carrying capacity | |  |
| Kamsarmax | Bulk carrier ship equal to 75-85k tonnes deadweight cargo carrying capacity, having a length of 229m to berth at the Port of Kamsar, a major bauxite shipping port in the Republic of Guinea | |  |
| LR1 | | Long Range tanker ship carrying capacity of 70k-75k tons | |
| LR2 | | Long Range tanker ship carrying capacity of 90k-110k tons | |
| MR tanker | Crude oil tanker ship size equal to 25-45k tonnes deadweight carrying capacity | |  |
| Panamax | Bulk carrier ship equal to 65-75k tonnes deadweight cargo carrying capacity, max size of bulk carrier ship to sail through the Panama Canal | |  |
| Suezmax | Crude oil tanker ship size equal to 120-160k tonnes deadweight carrying capacity, max size of tanker ship to sail through the Suez Canal | |  |
| VLCC | Very Large Crude Carrier tanker ship with carrying capacity of 250k-320k tons | |  |

# Appendix C – Definitions of shipping company roles and departments

| Shipping company role/department | Definition |
| --- | --- |
| Board of directors | Suggests and follows the company’s policy to all departments and co-ordinates all activities in the operation and management of the vessels as per company’s stated policies and objectives |
| Chartering department | Manage and follow up the procedures with charterers employing/hiring company’s vessels |
| Managing director | to achieve and implement the company’s policies and objectives |
| Performance department | work in conjunction with other technical staff to achieve the department’s stated principals, objectives and procedures |
| New building department | Managing and following up the company’s programme for ordering and fulfilling the building of new vessels at various shipyards |
| PMS department | Planned Maintenance System department – providing IT support in relation to all technical aspects of a ship |
| Bunkering department | The provision and monitoring of fuel oil and lubricants for the ships efficient operation |
| IT department | Onboard and onshore IT support |
| HR department | Recruiting and managing the selection and engagement of all shore-based personnel in accordance with the SMS procedures |
| Training department | Follow up the training requirements and needs as addressed by the company management system and prepare the training plan of shore and shipboard personnel |
| Insurance department | Ships hull and machinery and associated to Protection and Indemnity (P&I) for all vessels |
| DPA | Designated Person Ashore - act as a link between the vessel and the highest level of company management |
| HSQE | Health, Safety, Quality, Environment. The HSQE Department is the focal point within the Company for the management of all the health, safety, quality, energy and environmental (HSQEE) aspects of the vessels |
| Vetting department | Coordinating the vessels clearance by the vetting processes of Oil Majors (oil cargo charterers). Arrange, attend and respond to OCIMF Ship Inspection Report Exchange (SIRE) Program. Supervising the investigation of ship inspection reports contents and their corresponding corrective/preventive actions. Monitoring the implementation of corrective actions at ship/fleet level |
| Operations department | Responsible for ships operations and their proper performance in accordance to their commercial and legal obligations. Also, ensure that charterers expectations are fulfilled |
| Crew department | Recruitment and selection of competent and fully qualified senior and junior officers and ratings |
| SSO department | Shipboard Safety Organisation – to manage and follow up the on board the vessels stated principles, objectives and procedures as provide by regulatory authorities and the company SMS manual |
| Technical department | Responsible for all technical matters related to the performance and maintenance of each vessel in such a way, that the vessels equipment and machinery are best protected from damages and abnormalities and operated safely, efficiently and in compliance with all mandatory rules and regulations. |
| Technical director | To accomplish and comply with the technical and purchasing departments stated principles, objectives and procedures |
| Purchasing manager | Arranging the purchasing of all company’s goods and services for the ships and the headquarters in an efficient and cost effective manner |
| Fleet manager | To realise and comply with the technical department stated principles, objectives and procedures |
